# Electrospun Filaments Embedding Bioactive Glass Particles with Ion Release and Enhanced Mineralization

**DOI:** 10.3390/nano9020182

**Published:** 2019-02-01

**Authors:** Francesca Serio, Marta Miola, Enrica Vernè, Dario Pisignano, Aldo R. Boccaccini, Liliana Liverani

**Affiliations:** 1 Dipartimento di Matematica e Fisica “Ennio De Giorgi,” Università del Salento, Via Arnesano, I-73100 Lecce, Italy; francescaserio@unisalento.it; 2 Istituto Microelettronica e Microsistemi-CNR, Via Monteroni, Campus Unisalento, I-73100 Lecce, Italy; 3 Applied Science and Technology Department, Politecnico di Torino, Corso Duca degli Abruzzi 24, I-10129 Torino, Italy; marta.miola@polito.it (M.M.); enrica.verne@polito.it (E.V.); 4 Dipartimento di Fisica, Università di Pisa, Largo B. Pontecorvo 3, I-56127 Pisa, Italy; dario.pisignano@unipi.it; 5 NEST, Istituto Nanoscienze-CNR, Piazza S. Silvestro 12, I-56127 Pisa, Italy; 6 Institute of Biomaterials, Department of Materials Science and Engineering, University of Erlangen-Nuremberg, Cauerstr. 6, D-91058 Erlangen, Germany; aldo.boccaccini@fau.de

**Keywords:** poly(lactic acid) (PLLA), bioactive glass, scaffolds, electrospinning, composite fibres

## Abstract

Efforts in tissue engineering aim at creating scaffolds that mimic the physiological environment with its structural, topographical and mechanical properties for restoring the function of damaged tissue. In this study we introduce composite fibres made by a biodegradable poly(lactic acid) (PLLA) matrix embedding bioactive silica-based glass particles (SBA2). Electrospinning is performed to achieve porous PLLA filaments with uniform dispersion of bioactive glass powder. The obtained composite fibres show in aligned arrays significantly increased elastic modulus compared with that of neat polymer fibres during uniaxial tensile stress. Additionally, the SBA2 bioactivity is preserved upon encapsulation as highlighted by the promoted deposition of hydroxycarbonate apatite (HCA) upon immersion in simulated body fluid solutions. HCA formation is sequential to earlier processes of polymer erosion and ion release leading to acidification of the surrounding solution environment. These findings suggest PLLA-SBA2 fibres as a composite, multifunctional system which might be appealing for both bone and soft tissue engineering applications.

## 1. Introduction

Bone tissue continuously undergoes shape remodelling and repair at the microscale through processes of local regeneration, which are regulated by growth factors, hormones and the action of mechanical stresses. In reconstructive surgeries, the regeneration of bone and cartilage by autologous cell transplantation after an injury or tumour removal is one of the most promising strategies in order to reduce issues related to immunocompatibility and consequent immune rejection, as well as to avoid potential pathogen transfer [1]. However, since autologous grafts are often poorly available, an appealing approach is represented by the development of scaffold-based tissue engineering approaches based on bioactive materials for restoring bone morphology and function [2]. 

Therefore, enormous efforts are being made to create engineered constructs that mimic the physiological environment with its structural, topographical and mechanical properties. To this aim, various biomaterials, metals, natural or synthetic polymers and ceramics, have been investigated but no single one was proved to show all the crucial features required for an optimal scaffold [3,4]. In this framework, a promising approach relies on composite biomaterials with osteoconductive and osteoinductive capabilities, which might allow for osteogenesis stimulation while mimicking the extracellular matrix (ECM) morphology [4,5]. Several studies have been focused on the addition of inorganic and bioactive fillers, such as bioactive glasses, bioceramics or hydroxyapatite [6] in polymeric constructs, with the aim to promote chemical links to bone tissue by forming hydroxycarbonate apatite (HCA) layers as a result of ion leaching, in case of bioactive glass components, into the surrounding physiological fluids. In this process, the precipitation of microcrystalline HCA onto the scaffold surface [7] is due to a well-defined ion exchange mechanism between modifier ions (Na^+^ and Ca^2+^) in the glass and hydronium ions (H_3_O^+^) in the surrounding fluid, thereby causing dissolution of the glass network [8,9]. Compared to other bioactive materials, silica-based bioactive glasses are available in different compositions, which exhibit remarkable osteoinductive behaviour since they feature ionic dissolution products (Si^4+^, Mg^2+^, Ca^2+^) able to stimulate osteogenesis and angiogenesis [8,9]. In fact, the potential angiogenic effects of silica-based bioactive glass has been recently highlighted, through increased secretion of vascular endothelial growth factor involved in vascularization processes [10]. For these reasons, these materials are not only useful for bone tissue engineering but they might also promote the regeneration of soft tissues as needed in wound healing [11].

In fact, 45S5 Bioglass® has been largely used as inorganic phase in polymer foams and matrices to realize porous composites [12,13,14,15,16]. Silicate [17], borate [18] and phosphate-based glasses [19,20] have been recently tested in bulky bioresorbable polymeric sponges or polymer-coated scaffolds. A number of nano- and micro-fabrication technologies allow these systems to be processed as biocompatible and biodegradable fillers in polymers retaining higher surface-to-volume ratio and interconnected porous networks to better support tissue ingrowth and vascularization. In the last years, the electrospinning technology has been largely developed and notable progress has been made to realize biomimetic porous scaffolds designed for tissue engineering and for drug delivery [21,22,23]. Electrospinning is a versatile technique, which allows for the fabrication of polymer, ceramic or nanocomposite fibres with diameter ranging from a few tens of nanometres to a few micrometres, which strongly resemble the morphology of the native ECM and provide a networked architecture suitable for cell attachment [22,23,24,25]. Various different structures, morphologies and compositions can be achieved in fibres, to make them suitable for different tissue applications including vascular, bone, neural and tendon or ligament [21,25,26]. Particularly, fibres with nanocomposite materials and complex internal [27] or surface [28,29] nanostructures can be electrospun by blends of polymers or from colloidal solutions [30,31]. 

In this work we introduce nanocomposite electrospun fibres embedding silica-based bioactive glass (SBA2). SBA2 belongs to the SiO_2_–Na_2_O–CaO–P_2_O_5_–B_2_O_3_–Al_2_O_3_ class of systems, previously investigated as component of antibacterial and bioactive, bulky bone cements [7,32,33]. The FDA-approved polymer, poly(lactic acid) (PLLA), is chosen as matrix because of its excellent biocompatibility and biodegradability, already assessed in clinical treatments [1], as well as for its excellent processability with electrospinning [34]. The obtained PLLA-SBA2 fibres are characterized in their morphology and in their chemical and mechanical properties. The addition of inorganic particles in a polymeric matrix [35,36,37,38] leads to composites with varied mechanical properties, depending on the filler size and on their dispersion in the organic phase [35,36,38], as well as on the fabrication parameters, including the solvent used for electrospinning [38]. Additionally, acellular, in vitro bioactivity and cell viability are investigated, evidencing the biocompatibility of the PLLA-SBA2 fibrous composites. Overall, dispersed silica-based bioactive glass in resorbable polymeric composites with microscale texturing are highly promising systems for supporting cell cultures as well as for the development of biomedical applications. The novelty of this work is represented by the successful incorporation of SBA2 in electrospun PLLA fibres, not reported previously in literature and also on the correlation of the nanopores on the fibres surface with fibres degradation and bioactive glass particles release.

## 2. Materials and Methods 

### 2.1. Bioactive Glass Synthesis and Characterization

The SBA2 glass has the following nominal composition (mol %): 48% SiO2, 18% Na_2_O, 30% CaO, 3% P2O5, 0.43% B2O3, 0.57% Al_2_O_3_ and was synthesised by melt-quenching route, as detailed elsewhere [7,32,33]. Briefly, reagent-grade reactants [SiO_2_, Na_2_CO_3_, CaCO_3_, Ca_3_(PO_4_)_2_, H_3_BO_3_ and Al_2_O_3_] were melted in a platinum crucible at 1450 °C for 1 h (Carbolite HTF 1800, CARBOLITE GERO, Neuhausen, Germany), the melt was then quenched in water to obtain a frit. The frit was ball milled in aqueous medium. The grain size distribution of milled SBA2 was estimated using a particle size analyser (Sympatec Helos H0621, kindly performed at CERICOL Research Centre, Sovigliana, Vinci (Firenze), Italy); the specific surface area (SSA) of milled SBA2 was assessed by using the Brunauer-Emmet-Teller (BET, ASAP2020Plus-Micromeritics, Aachen, Germany) method [39]. The obtained glass powder was analysed morphologically and compositionally by scanning electron microscopy (SEM, FEI QUANTA INSPECT 200, Eindhoven, The Netherlands) and energy dispersive X-ray spectrometry (EDS) (EDAX PV 9900, Weiterstadt, Germany). Thermal properties were determined by differential thermal analysis (DTA–404 PC instrument, Netzsch, Selb, Germany), in a temperature range of 20–1300 °C, using a heating rate of 10 °C/min and high-purity alumina as reference.

### 2.2. Electrospinning

PLLA (molecular weight 85−160 kg mol^−1^, Sigma-Aldrich, Munich, Germany) was dissolved in a mixture of dichloromethane and acetone (80:20 *v*/*v*) at a concentration of 20% (*w*/*v*) at room temperature. The solution was stirred overnight, then SBA2 was added with a concentration of 7% *w*/*v*. The resulting suspension was vigorously mixed and then stirred again overnight to obtain a homogeneous dispersion of SBA2 in the polymer solution. The solution was put in an ultrasound bath for 1 hour prior the electrospinning with the aim to reduce SBA2 clustering and then transferred in a 1 mL syringe. The spinning process was performed by a 21G stainless steel needle and a syringe pump (Harvard Apparatus, Holliston, MA, United States) with feeding rate 0.8 mL h^−1^ and by applying a positive high-voltage of 12 kV (EL60R0.6-22, Glassman High Voltage, XP Power, Milano, Italy) between the needle and a metal collector. Random fibre mats were collected on a static grounded 10 × 10 cm^2^ collector meanwhile aligned fibre mats were collected on a grounded disk (8 cm diameter, 1 cm thickness) rotating at a speed of 5000 rpm, maintaining constant all the other processing parameters. The air relative humidity and temperature during the electrospinning process were about 40% and 20°C, respectively. The needle-collector distance was adjusted to 15 cm. Neat PLLA fibres were fabricated as reference material by using identical set-up parameters. 

### 2.3. Morphology and Mechanical Properties of PLLA-SBA2 Fibres

PLLA-SBA2 electrospun fibres were sputtered with gold by using a Sputter Coater (Q150T, Quorum Technologies, Darmstadt, Germany) and then inspected by SEM and energy dispersive X-ray spectrometry (EDS) (Auriga 0750, ZEISS, Jena, Germany). Average fibre diameters were calculated by analysing a total of at least 100 fibres for each sample, using the software ImageJ [40]. Regarding mechanical properties, as reported by Ricotti et al. [41], mechanical differences in uniaxial aligned arrays are more pronounced. Thus, aligned fibre mats were used in this paper to investigate more specifically the mechanical properties of PLLA-SBA2 composite fibre mats. Mechanical properties of random and aligned composite fibres mats at room temperature were investigated by uniaxial tensile strength tests using a universal testing machine (K. Frank GmbH, Mannheim, Germany). Each sample was cut into a rectangular shape (with cross-section area of 5 × 4 mm^2^) using a paper square framework and its thickness is measured by using a digital micrometre (0.02–0.06 mm). Then measurements were carried out at a crosshead speed of 10 mm/min using a 50 N load cell, according to a previous study [42] and the resulted stress-strain curves are used to obtain Young’s modulus, elongation at break and tensile strength.

### 2.4. Degradation studies

The degradation behaviour of composite fibres was investigated by immersion in Dulbecco’s phosphate buffered saline (DPBS, Sigma-Aldrich, Munich, Germany) medium at pH 7.4. Resulting pH values were measured instantly after the immersion of the sample and for different time points, up to 21 days, at 37 °C by using a pH meter (HD8705, Delta OHM, Padova, Italy). The pH measurements of different solutions were correlated to the dissolution of ions in the incubation media. In addition, the degradation of PLLA-SBA2 fibres in physiological solutions was assessed by measuring the weight loss [23] for dried fibrous mats after 21 days of DPBS incubation. The percentage of weight loss, *W*_L_ %, was computed as 100 × (*W*_0_ − *W*_r_)/*W*_0_, where *W*_0_ and *W*_r_ are the initial and the residual weight of the sample, respectively.

### 2.5. Acellular Bioactivity

The acellular bioactivity of PLLA-SBA2 fibres, related to the surface deposition of HCA layers, was evaluated by immersing the samples inserted in scaffold holders (CellCrownTM 24, Scaffdex, Sigma Aldrich, Munich, Germany) in simulated body fluid (SBF) solution [43] for 1, 7, 14 and 21 days at 37 °C, on an oscillating tray in an incubator. A falcon tube with only SBF was used as control. After each time point, samples were washed with distilled water, dried at room temperature before SEM-EDS characterization and Fourier Transform Infrared Spectroscopy (FTIR) analysis in attenuated total reflectance (ATR) mode. PLLA neat fibres were considered as control. FTIR spectra were recorded by a spectrometer (IRAffinity-1S, Shimadzu, Kyoto, Japan), repeating 32 scans over the wavenumber range 4000–500 cm^−1^, with a resolution of 4 cm^−1^. 

### 2.6. Cell Cultures

PLLA-SBA2 fibres were disinfected under an ultraviolet lamp for 1 h. Murine-derived stromal cells ST-2 (obtained from Leibniz-Institut DSMZ-Deutsche Sammlung von Mikroorganismen und Zellkulturen GmbH, Braunschweig, Germany), were cultured to confluence in 75 cm^2^ culture flasks in Roswell Park Memorial Institute medium (RPMI 1640) (GibcoTM, Thermo Fisher Scientific, Schwerte, Germany) containing 10% foetal bovine serum (FBS; Lonza) and 1% penicillin/streptomycin (Lonza) at 37 °C and 5 % CO_2_. Before seeding, cells were detached using Trypsin in DPBS (Sigma Aldrich, Munich, Germany), stained with 0.4% (*v*/*v*) trypan blue solution and counted using a Neubauer chamber (VWR). Then, ST-2 cells were seeded onto the electrospun scaffolds (including neat PLLA fibres as control) with a density of 2 × 10^4^ cells/cm^2^. All samples were assayed in triplicate and each sample was incubated in the same RPMI medium described above. Cells were cultured for 7 days renewing the culture medium once after 3 days of culture. Viability analyses of ST-2 cells on composite fibres was assessed after a cultivation period of 1 day and 7 days by using a WST-8 assay (Cell Counting Kit-8, Sigma Aldrich, Munich, Germany), which is based on the conversion of tetrazolium salt to highly water-soluble formazan by mitochondrial enzymes of viable cells. At each time point, the culture medium was removed from each sample and each well with samples and cells was washed with DPBS and added with a solution of 10% WST-8 reagent in colourless medium. After an incubation period of 3 hours at 37 °C and 5% CO_2_, the solution was transferred into a 96 well plate to measure absorbance at 450 nm by using a microplate Elisa reader (PHOmo Elisa reader, Autobio Diagnostics Co. Ltd., Zhengzhou, China). 

To investigate cell morphology, a preliminary evaluation was provided by SEM analysis after 1 day and 7 days of culture. Samples were fixed by using a solution containing paraformaldehyde, glutaraldehyde, sodium cacodylate trihydrate and sucrose (Sigma Aldrich, Munich, Germany). Subsequently, samples dehydration was achieved by using a series of aqueous ethanol solutions. The samples were then dried in a critical point drier (Leica EM CPD 300, Leica, Wetzlar, Germany) and sputtered with gold. The cytoskeleton organization and nucleus morphology of cells on PLLA-SBA2 fibres were studied after 1 day and 7 days following seeding by staining with rhodamine phalloidin and DAPI (ThermoFisher Scientific, Schwerte, Germany). Briefly, samples were fixed by using a fixation solution containing 1,4-piperazinediethanesulfonic acid buffer, ethylene glycol tetraacetic acid, polyethylene glycol, paraformaldehyde, DPBS and sodium hydroxide (Sigma Aldrich, Munich, Germany), washed with DPBS and immersed in a permeabilization buffer for intracellular staining. 400 µL of a 8 µL/mL DPBS solution of rhodamine phalloidin was added in each well containing samples, then kept for 1 hour at 37°C. After the removal of the dye, samples were vigorously washed with DPBS and 400 µL of a 1 µL/mL DPBS solution of DAPI was added to each well. Then, samples were washed in DPBS and analysed with a fluorescent microscope (Axio Scope A1, Zeiss, Jena, Germany). 

### 2.7. Statistical Analysis

Each experiment was repeated three times. All results of cell viability and average fibre diameter were expressed as (mean ± standard deviation) and a one-way analysis of variance (ANOVA) was used for statistical analysis. A P value < 0.05 was considered statistically significant. 

## 3. Results and Discussion

The BET analysis evidenced a SSA of 11.6 m^2^/g, the particle size analysis showed a non-symmetric distribution with a mode grain size of 2 μm; characteristic particle sizes are *d*_50_ = 2.0 µm and *d*_90_ = 4.4 µm, with 94% of grain sizes below 5 µm and the residual 4% below 9 µm.

**Figure 1:**
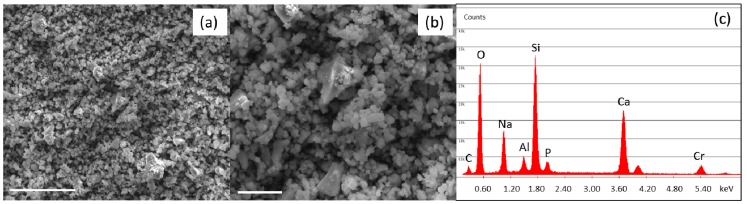
Scanning electron microscopy (SEM) (**a**,**b**) and energy dispersive x-ray spectrometry (EDS) (**c**) analysis of SBA2 powders after milling process. Scale bars: 40 µm (**a**), 10 µm (**b**).

Figure 1 shows the morphology (a, b) and the compositional analysis (c) of SBA2 glass powders. The powders display the typical sharp-cornered morphology of ball-milled glass. Moreover, as previously mentioned, the majority of SBA2 powders showed a grain size < 5 µm. In addition, the performed DTA analysis evidenced a glass transition temperature of about 550 °C, the crystallization onset at 600 °C, a crystallization peak at 655 °C and a melting temperature of 1220 °C.

**Figure 2:**
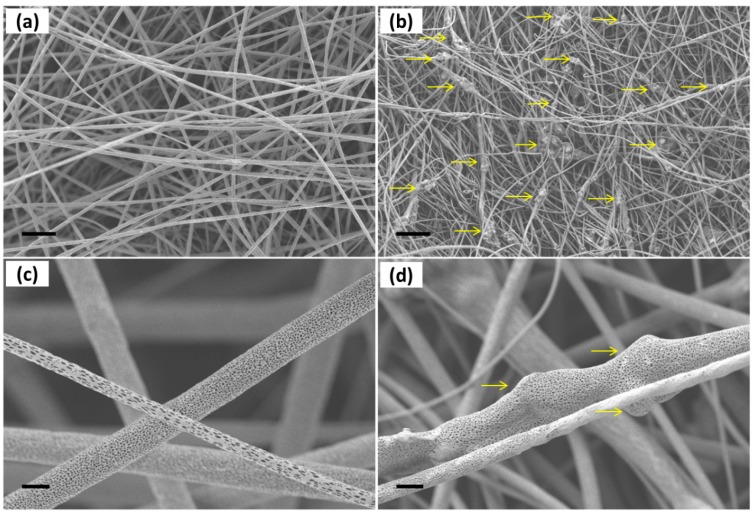
SEM micrographs of (**a**) neat polylactic acid (PLLA) fibres and (**b**) PLLA-SBA2 fibres (scale bar = 25 µm). Neat PLLA fibres (**c**) and composite fibres (**d**) at high magnification, evidencing their highly porous surface. Scale bar: 2 µm.

The electrospinning process of PLLA-SBA2 is carried out by adjusting the voltage bias as well as the solution flow rate to obtain almost bead-free fibres. The morphology of neat PLLA and composite fibres is shown in SEM micrographs in Figure 2. Composite fibres exhibit a quite homogeneous distribution of embedded SBA2 particles (arrows in Figure 2b).

**Figure 3:**
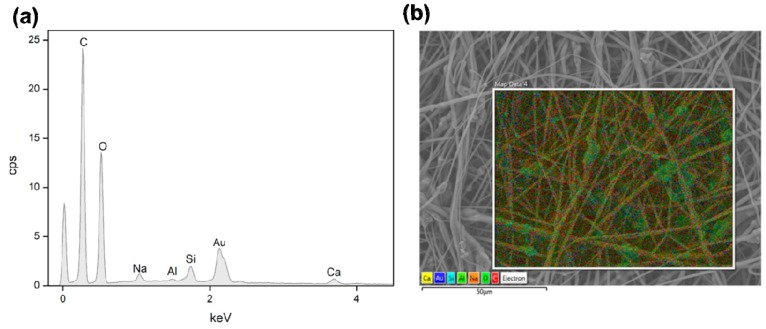
EDS spectrum of electrospun PLLA-SBA2 fibres (**a**) and its comparative SEM-EDS layered image (**b**) showing peaks of SBA2 constituents such as Ca (yellow), Na (orange) and Si (cyan). O (bright green) and C (red) are mainly related to PLLA.

The SBA2 incorporation also leads to an increase of the overall roughness in the electrospun mats. Interestingly, the average diameter of PLLA-SBA2 filaments is around one half of fibres without SBA2 (Table 1), similarly to previous findings on composite fibres made by PCL and commercially available Bioglass® (45S5) particles [42]. This effect might be explained due to the change of intrinsic solution properties, including rheology and conductivity, of the polymeric solutions after the addition of SBA2 particles [44]. In addition, the physical effect of particles in the electrified solution is associated to the onset of enhanced whipping and varicose instabilities, which affect the morphology of the ultimately deposited fibres [45], as shown by the local increase of filament radius in the composite fibres close to the particles. The resulting excess mass due to local polymer accumulation leads to the here found decrease of diameter along the rest of the fibre length due to the overall polymer mass conservation. Furthermore, we find that both the types of fibres display cylindrical shape and high surface porosity, with pore size around 100 nm (Figure 2c,d), which is characteristic of PLLA electrospun with highly volatile solvents [28] and expected to favour cell attachment. Indeed, it is known that both micro- and nanoporosity play an important role in protein adhesion and cell function [46]. In addition, the incorporation of the bioactive glass particles in PLLA-SBA2 fibres is confirmed by SEM-EDS analysis as shown in Figure 3 and Figure S1. 

**Figure 4:**
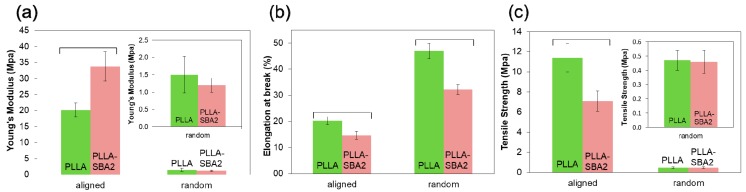
Young’s modulus (**a**), elongation at break (**b**) and tensile strength (**c**) of random and aligned fibres. Results are expressed as (mean ± standard deviation). Bars show statistically significant differences (*p* < 0.05). In the inset of (**a**) and (**c**) a zoom view of the properties of randomly oriented fibres is reported.

The mechanical properties of the polymeric and composite fibres mats, determined from their stress–strain curves, are summarized in Figure 4, in terms of Young’s modulus (Figure 4a), elongation at break (Figure 4b) and tensile strength (Figure 4c) for either randomly oriented and aligned electrospun samples. Particularly, in uniaxially aligned arrays, in which the fibres and to some extent polymeric chains [47,48,49,50,51] are oriented along the traction axis, mechanical differences between composite and neat PLLA are more pronounced [49]. The elastic modulus of PLLA-SBA2 fibres is found to be (34 ± 5) MPa, significantly higher than the value measured for pristine PLLA fibres, (20 ± 2) MPa, just for the aligned fibres. Correspondingly, PLLA-SBA2 exhibits a relatively lower elongation at break (15 ± 2%) compared to the value of PLLA samples in the same alignment conditions (20 ± 2%), as well as higher tensile strength (11 ± 1 MPa), compared to (7 ± 1) MPa. The mechanical behaviour of the composites tightly depends on the interaction at the polymer-bioactive glass interface and on the homogeneity of the dispersion of glass particles in the fibres, which if poor would lead to agglomeration in clusters [51]. The present findings in terms of increased elasticity and concomitant reduction of the elongation at break and tensile strength are indicative of a reinforced fibre system where inorganic fillers lead to embrittlement of the fibres [39,52].

**Figure 5:**
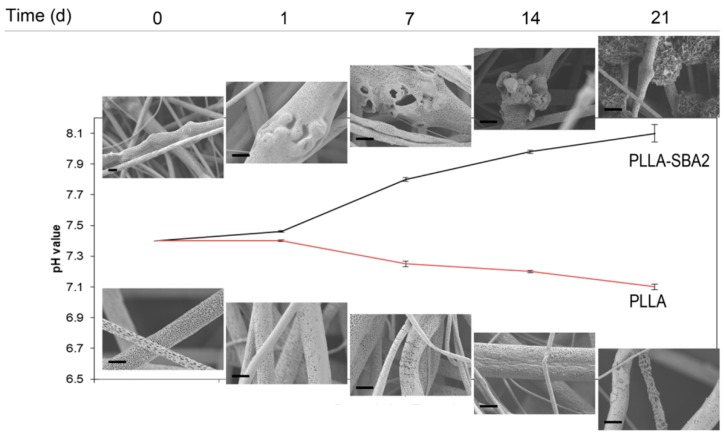
In vitro degradation studies and dissolution of PLLA and PLLA-SBA2 electrospun fibres. Change in the pH of DPBS solution for PLLA and PLLA-SBA2 fibres, which is associated to the acid hydrolysis of the polymer components and to the release of SBA2 into the solutions at 0, 1, 7, 14, 21 days from incubation and the relatives SEM micrographs at high magnification of composite and polymeric fibres along soaking experiments in SBF at the same time point (scale bar = 2 µm).

The analysis of both PLLA-SBA2 and PLLA fibres, performed by collecting SEM micrographs of samples soaked in SBF solution, evidences polymer degradation starting from the 7th day following incubation, resulting in cracks and points of break in the surface of fibres. As highlighted in SEM micrographs in the top of Figure 5, in PLLA-SBA2 fibres this degradation leads to a progressively increasing exposure of glass particles to the surrounding microenvironment. In order to investigate the resulting bioactive glass dissolution from PLLA cracks propagating along the nanopores on the fibre surface, we perform further in vitro degradation studies, incubating the composite scaffolds in DPBS in physiological conditions (at pH = 7.4 and 37 °C), as showed in Figure S2c. During the immersion in DPBS for 21 days, the corresponding changes of the solution pH was monitored, as displayed in Figure 5 together with corresponding SEM micrographs at each time point. PLLA fibres clearly undergo acid hydrolysis in the solution which leads to a decrease of pH, whereas for the composite fibres this trend is overcome by the release of SBA2 dissolution products into the solution. The pH increase related to bioactive glass dissolution products have been already investigated in several medium by Cerutti et al. [52]. This increased pH associated with the dissolution-precipitation of bioactive glass is known to affect various cellular processes, being correlated with increased metabolic activity and proliferation rate in mammalian cells [53]. Our data on pH variations are also supported by the corresponding measurements of sample weight loss, performed after 21 days, which is found to be 2.2% of the initial weight for neat PLLA fibres and 9.7% for PLLA-SBA2 fibres.

**Figure 6:**
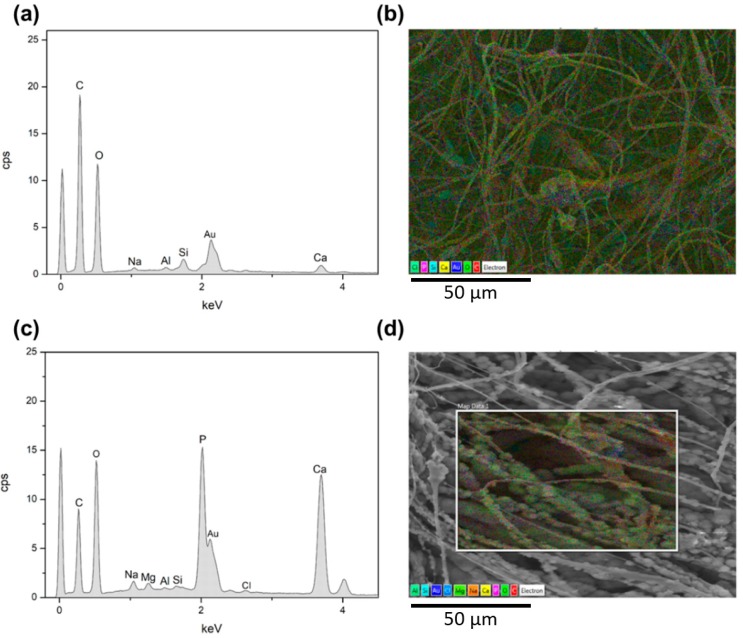
EDS analysis of PLLA-SBA2 samples after 7 days (**a**,**b**) and 21 days (**c**,**d**) of soaking in SBF. EDS spectra of PLLA-SBA2 (**a**,**c**) and comparative EDS layered images (**b**,**d**) reveal an increment of Ca (yellow) and P (purple) content. SBA2 constituents: Na (orange), Al (green) and Si (cyan). O (bright green) and C (red) mainly relate to PLLA.

**Figure 7:**
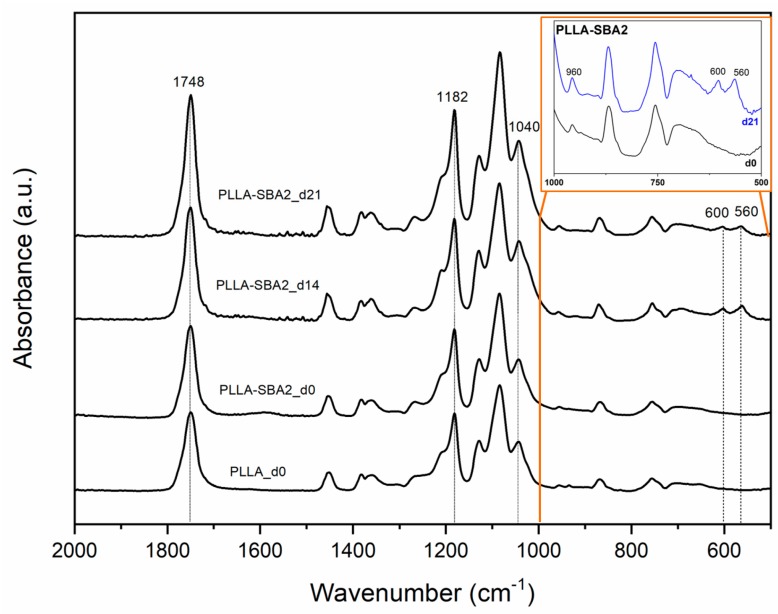
Fourier transform infrared (FTIR) spectra of neat PLLA (PLLA_d0) and composite scaffolds (PLLA-SBA2_d0) before incubation in simulated body fluid (SBF) and of composite fibres after 14 (PLLA-SBA2_d14) and 21 days (PLLA-SBA2_d21) of incubation in SBF. The inset better highlights the new peaks ascribable to hydroxycarbonate apatite (HCA) formation mentioned in the text.

The acellular bioactivity of the electrospun fibres is evaluated by exploring the formation of HCA on their surfaces upon soaking in SBF, at 37 °C for different time periods. The bioactivity of PLLA-SBA2 fibres can be clearly appreciated when a large quantity of HCA with typical cauliflower-like morphology is found nearby the electrospun filaments (rightmost SEM micrographs in the top of Figure 5). Neat fibres, instead, do not lead to HCA formation. These findings are supported by EDS, detecting high Ca and P peaks belonging to HCA (Figure 6) with Ca/P ratio of 1.9, as well as by FTIR analysis (Figure 7). Indeed, FTIR spectra of PLLA-SBA2 fibres following incubation feature two new peaks at 600 cm^−1^ and 560 cm^−1^ corresponding to HCA-associated P–O groups asymmetric bending [54] and the increase in intensity of the peak centred at 960 cm^−1^ ascribable to the contribution of Si-OH symmetric stretching characteristic of the bioactive glass after immersion in SBF [54,55].

The formation of HCA following immersion in SBF solution confirms that the incorporation of the bioactive glass particles in the PLLA filaments is not preventing the characteristic bioactivity of this specific composition of bioactive glass to be highlighted [32]. In addition, the formation of HCA after 21 days suggests this class of scaffolds not only as useful systems for bone tissue engineering but also for soft tissue repair applications, because release of ions is detected since the earliest time point, as evidenced by the pH variation in the degradation studies. In particular, these fibrous architectures would be highly suitable for the release of therapeutic ions embedded in the bioactive glass structure, occurring without simultaneous damage in the fibrous structure of the scaffolds. Indeed, the double-scale temporal dynamics exhibited by the two mechanisms might be highly advantageous for wound healing processes, where the composite can still provide mechanical support while serving as reservoir of therapeutic ions [56]. The ability to modulate ion release through bioactive glass composition engineering, in relation to the addressed cell type, might be especially important in this respect. This relevance is also suggested by previous works that emphasized the influence of ion release in stimulating vascularization and triggering the production of angiogenic growth factors during soft tissue repair [57,58]. Other application fields potentially benefiting from interplaying ionic release and gradual morphological changes in composite scaffolds include the development of biomedical materials able to modulate the inflammatory response [59], to control cell proliferation [11], to support the regeneration of the peripheral nerve and the treatment of relevant pathological conditions such as chronic osteomyelitis [60,61,62]. The smart combination of biologically active ion release and nanotopography has been highlighted for tissue engineering [63].

**Figure 8:**
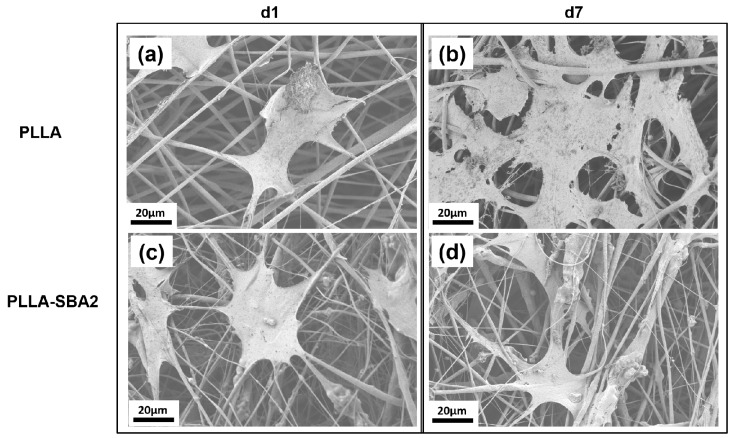
SEM micrographs of dehydrated ST2 cells, cultured on neat (**a**,**b**) and composite (**c**,**d**) fibres for 1 (**a**,**c**) and 7 (**b**,**d**) days. Scale bar: 20 μm.

**Figure 9:**
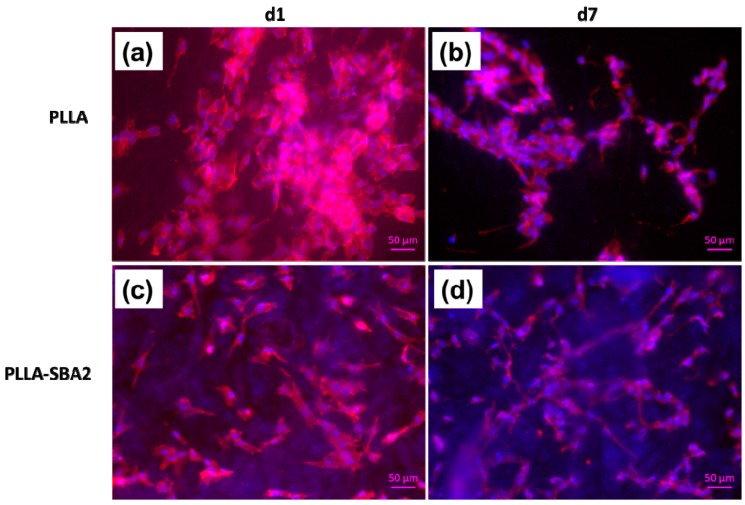
Fluorescent labelled actin filaments (red) and nuclei (blue) in ST2 cells cultured on neat PLLA (**a**,**b**) and PLLA-SBA2 (**c**,**d**) fibres, after 1 day (**a**,**c**) and 7 days from cell seeding. Scale bar: 50 μm.

**Figure 10:**
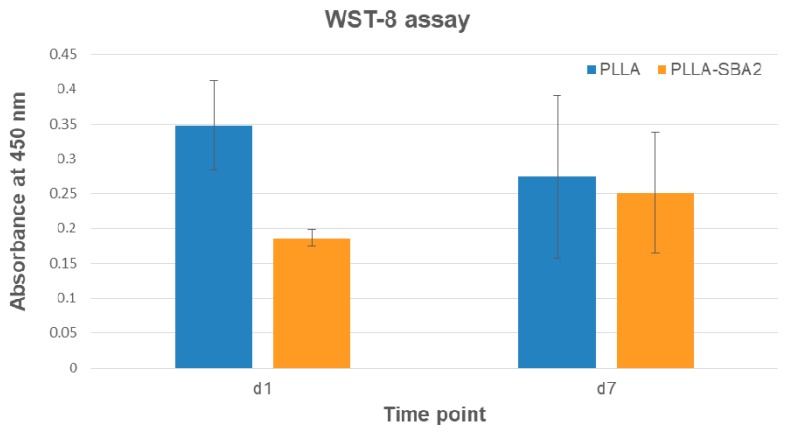
WST-8 assay: histograms of absorbance at 450 nm for PLLA and PLLA-SBA2, one (d1) and seven (d7) days after the seeding.

Finally, we investigated the biocompatibility of PLLA-SBA2 fibres using a murine-derived ST2 stromal cell line. The morphology of cells, inspected by SEM, was found to be affected by the fibrous structure of the scaffold, with significant cells elongation along the directions of supporting fibres as shown in Figure 8. The cytoskeleton actin and the nuclei of cultured cells were also analysed by immunofluorescence microscopy (Figure 9). Overall, cells adhesion was efficient on the nanocomposite filaments embedding bioactive glass particles. In previous works, in vitro cytotoxicity tests of SBA2 in PMMA-based cement were accomplished using the indirect contact method [33]. Here, the cell viability is directly evaluated at 1 and 7 days after seeding. WST-8 assays highlight, for absorbance detected at 450 nm at day 1 for cultures on PLLA fibres, an almost double value compared to those measured for cultures on PLLA-SBA2, results shown in Figure 10. This result correlates well with the increase of the pH value found during degradation studies, namely with the release of ions from the bioactive glass particles. Indeed, it is known that the alkalinisation of the medium can influence significantly cell metabolism [64] and can consequently induce distinct phases in cell cycling on composite and neat fibres, respectively, as also suggested by previous reports focused on the behaviour of human osteoblasts exposed to the ionic dissolution products of bioactive glass [65,66]. 

At day 7 after seeding the values of absorbance measured on PLLA fibres and on PLLA-SBA2 fibres become comparable, as shown in Figure 10, evidencing that the initial conditions of pH changes do not affect cell viability at longer timescales. ST2 cells cultured on composite fibres generally exhibit a relatively lower cell density following seeding, then reaching cell densities comparable to those on PLLA fibres after 7 days. Indeed, despite a significant difference in cell density immediately after cell seeding, these results evidence the ability of PLLA-SBA2 fibres to providing an effective and viable environment for subsequent scaffold colonization. 

## 4. Conclusions

This study investigated the potentiality of electrospun PLLA-SBA2 fibres as potential scaffold material for tissue engineering. The bioactive glass (SBA2) micro-sized powder was effectively incorporated in electrospun polymer filaments and the obtained composite system was characterized in terms of morphology and mechanical properties. The acellular bioactivity and biocompatibility of the PLLA-SBA2 fibres was assessed and HCA deposition after 21 days of immersion in SBF was found to be promoted by the embedded bioactive glass particles at a later stage compared with ion release, suggesting this system as multifunctional scaffold appealing for both bone and soft tissue engineering applications.

## Figures and Tables

**Table 1:** Average diameters, minimum and maximum values measured for the transversal size of neat polymeric and composite fibres.

Sample	Average Fibre Diameter (µm)	Minimum Fibre Transversal Size (µm)	Maximum Fibre Transversal Size (µm)
PLLA	2.0 ± 0.2	1.0	3.9
PLLA-SBA2	1.0 ± 0.2	0.3	2.5

## Supplementary Materials

The following are available online at http://www.mdpi.com/2079-4991/9/2/182/s1, Figure S1: EDS analysis performed on PLLA-SBA2 sample before (top) and after 21 days of immersion in SBF solution (bottom), Figure S2: SEM micrographs of neat PLLA fibers (a), composite PLLA-SBA2 fibers (b), composite fibers after 7 days in DPBS (c) and 21 days in SBF (d).
