# Supplementary material for: Electrospun Filaments Embedding Bioactive Glass Particles with Ion Release and Enhanced Mineralization
Source: Nanomaterials (Basel). 2019 Feb 1;9(2):182. doi: 10.3390/nano9020182 (PMC6410207; doi:10.3390/nano9020182)
Supplement: Supplementary file 1 [file nanomaterials-09-00182-s001.pdf]

Supplementary Materials

Electrospun filaments embedding bioactive glass particles with ion release and enhanced mineralization

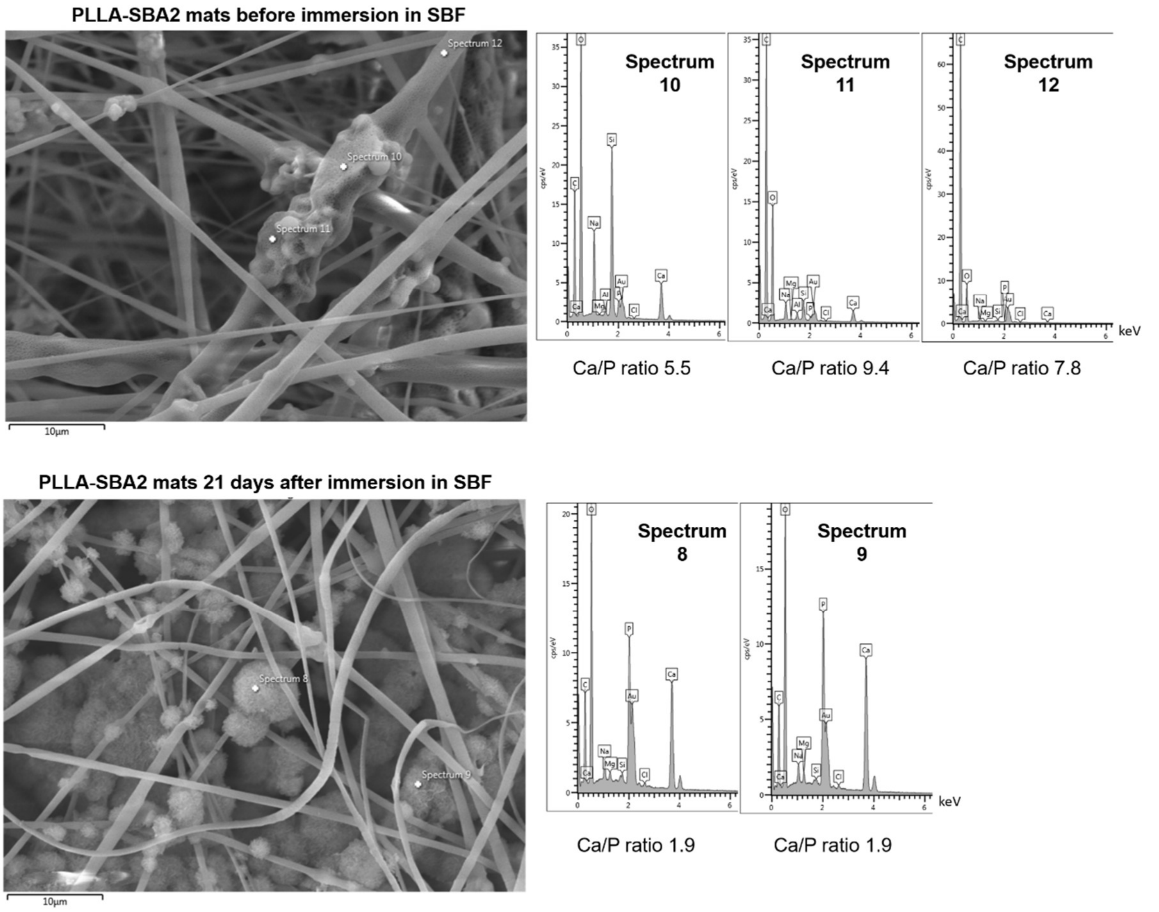

Figure S1. EDS analysis performed on PLLA-SBA2 sample before (top) and after 21 days of immersion in SBF solution (bottom).

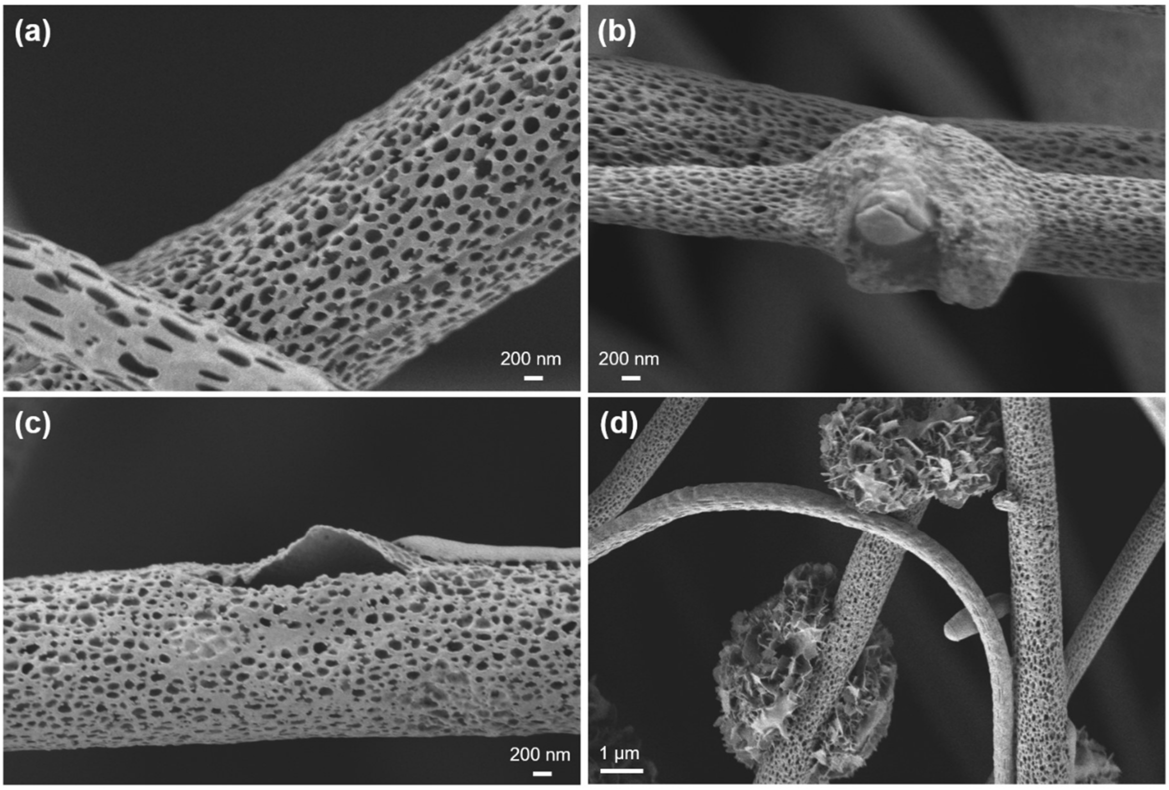

Figure S2. SEM micrographs of neat PLLA fibers (a), composite PLLA-SBA2 fibers (b), composite fibers after 7 days in PBS (c) and 21 days in SBF (d).
